# Density Functional Theory Study of the Trans-Trans-Cis (TTC)→Trans-Trans-Trans (TTT) Isomerization of a Photochromic Spiropyran Merocyanine

**DOI:** 10.3390/molecules13061246

**Published:** 2008-06-03

**Authors:** Grazia Cottone, Rosina Noto, Gianfranco La Manna

**Affiliations:** 1 Dipartimento di Scienze Fisiche ed Astronomiche, Università di Palermo, Via Archirafi 36, I-90123 Palermo and CNISM, Italy; E-mail: cottone@fisica.unipa.it; 2 IBF Sezione di Palermo, Via Ugo La Malfa 153, I-90146 Palermo, Italy; E-mail: rosina.noto@pa.ibf.cnr.it; 3 Dipartimento di Chimica Fisica "F. Accascina", Università degli Studi di Palermo, Viale delle Scienze, I-90128 Palermo, Italy

**Keywords:** Spiropyran, photochromism, DFT calculation, solvent influence on activation energy, merocyanine

## Abstract

Density Functional Theory (DFT) calculations have been performed on the TTC→TTT isomerization reaction of the open forms of the 1',3'-dihydro-8-bromo-6-nitro-1',3',3'-trimethylspiro[2H-1-benzopyran-2,2'-(2*H*)indole (8-Br-6-nitro-BIPS) system. The calculations were carried out *in vacuo* and in methylene chloride solution at different temperatures. Results are compared with the available experimental values of free energy difference and activation energy in solution.

## Introduction

Spirobenzopyrans (SP) are bistable photochromic molecules which are converted, upon ultraviolet excitation, from the closed, colourless form to an open coloured form (merocyanine, ME). Because of this behaviour, SP are considered suitable materials for a number of technological applications like optical memories [1,2,3], molecular switches [3,4,5,6], as well as models of biological receptors [7,8].

**Figure 1:**
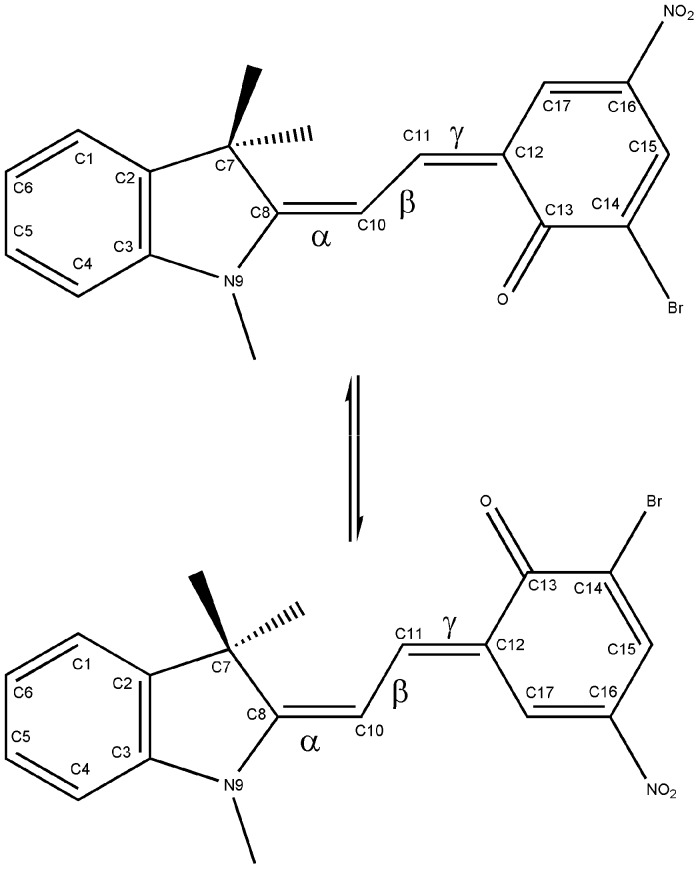
– Atom numbering of the TTC and TTT isomers.

In a previous paper [9] we analyzed the process of the thermal reaction SP⇆ME by performing quantum mechanical calculations at the Density Functional Theory (DFT) level on the ground state of the well-known nitro-substituted spirobenzopyran 1',3'-dihydro-6-nitro-1',3',3'-trimethylspiro[2H-1-benzopyran-2,2'-(2*H*)-indole] (6-nitro-BIPS). The results suggested that the ring-opening reaction proceeds through a multistep pathway, involving some conformers of the open merocyanine form. The ME conformers differ in the values of the three dihedral angles N9–C8–C10–C11 (α), C8–C10–C11–C12 (β) and C10–C11–C12–C13 (γ) (see Figure 1 for the atom numbering), so they are labelled with a three-letter code indicating the cis (C) or trans (T) value for the torsional angles α, β and γ, respectively. As already reported [10], the ME isomers having the β angle in the cis configuration are the least stable forms.

DFT calculations, performed at level of isolated molecules, showed that the most stable form of ME is the TTC one, whereas the other conformers are more energetic by values ranging from 5.8 kJmol^-1^ (TTT) to 72.8 kJmol^-1^ (TCT) (ΔG° values at room temperature).

Several studies have examined the role of substituents and the solvent on the mechanism of photochromism [5,11]. In particular, results from NMR experiments on the substituted SP 6-nitro-8-Br-BIPS (Figure 1) showed the existence of an isomeric distribution of merocyanines in the presence of solvent, involving the TTC and TTT isomers, the TTC being the dominant isomer, giving an evaluation of the free energy difference between the two isomers and of the activation energy of the isomerization reaction [12]. Since these data were obtained in the presence of solvent, we attempted to carry out a theoretical evaluation of them, in order to also understand the importance of considering the solvent when performing quantum-mechanical calculations.

In the present work, DFT calculations have been performed to study the isomerization reaction TTC→TTT of the open forms (merocyanines) of the 8-Br-6-nitro-BIPS system. Calculations were carried out at level of isolated molecules as well as in methylene chloride solution, at two different temperatures. Results are compared with the available experimental values of isomerization free energy and activation energy in methylene chloride solution at low temperature [12]. 

## Computational Methods

All the calculations were performed by using the DFT method (B3LYP parameterization) with the split-polarized 6-31G(d,p) basis set, with the GAUSSIAN03W software package [13]. The presence of the solvent was accounted for by using the conductor-like polarizable continuum model (C-PCM) [14,15]. The existence of the stationary points were checked by inspection of the hessian matrix eigenvalues. Vibrational frequency analysis in the harmonic approximation was also performed, and thermochemical data were obtained at 190 and 298 K. 

To locate the transition states, energy scanning as a function of the γ torsional angle was performed (see Figure 1 for the definition), while all other geometrical parameters were optimized; the obtained maxima were used as the starting geometries for further full optimizations as first-order saddle points. In order to obtain convergence, it was necessary to freeze the rotation of the three methyl groups. 

## Results and Discussion

### Isomerization energy

The energy values obtained for the systems TTT and TTC, both in *vacuo* and in methylene chloride solution, are reported in Table 1. 

The thermodynamic data were evaluated at 190 K in order to allow a direct comparison with the experimental values at the same temperature, as well as at room temperature. The theoretical free energy difference, ΔG, between the TTC and TTT isomers, evaluated *in vacuo* is 5.8 kJ/mol^-1^, at both temperatures. As shown in Table 1, the differences of the internal energy, enthalpy and free energy values, ΔE, ΔH and ΔG, respectively, evaluated at 190 K, decrease when considering the solvent, within the range 5%-15%. A further decreasing is observed when increasing the temperature only in the case of the free energy (from 5.3 kJ mol^-1^ at 190 K to 4.5 kJ mol^-1^ at 298 K in CH_2_Cl_2_ solution). Compared to the gas phase, the solvent has no effect on the values of the α, β and γ dihedral angles, whereas an increasing of the C8-C10 and C11-C12 bond lengths with a decreasing of C10-C11 bond length is observed in both isomers. The value of 5.3 kJ mol^-1^ obtained for the free energy difference at 190 K in solution is in good agreement with the experimental value, 4.6±0.7 kJ mol^-1^ [12].

**Table 1:** Relative energies (kJ·mol^-1^), relevant torsional angles (degrees) and bond lengths (Å) of 8-bromo-6-nitro-BIPS (TTC and TTT isomers) in *vacuo* and in methylene chloride solution at 190 K and 298 K.

	8-Br-6-NO_2_-BIPS *in vacuo*		8-Br-6-NO_2_-BIPS in CH_2_Cl_2_ solution	
	TTC	TTT	TTC	TTT
**E**	0.	6.8	0.	5.8
**H_190_**	0.	6.3	0.	6.0
**G_190_**	0.	5.8	0.	5.3
**H_298_**	0.	6.6	0.	6.0
**G_298_**	0.	5.8	0.	4.5
**α**	180.	180.	180.	180.
**β**	180.	180.	-179.9	180.
**γ**	0.	180.	0.1	180.
**C8-C10**	1.390	1.388	1.407	1.405
**C10-C11**	1.398	1.400	1. 384	1.383
**C11-C12**	1.406	1.398	1.424	1.418

### Transition states structures and activation energy

**Figure 2:**
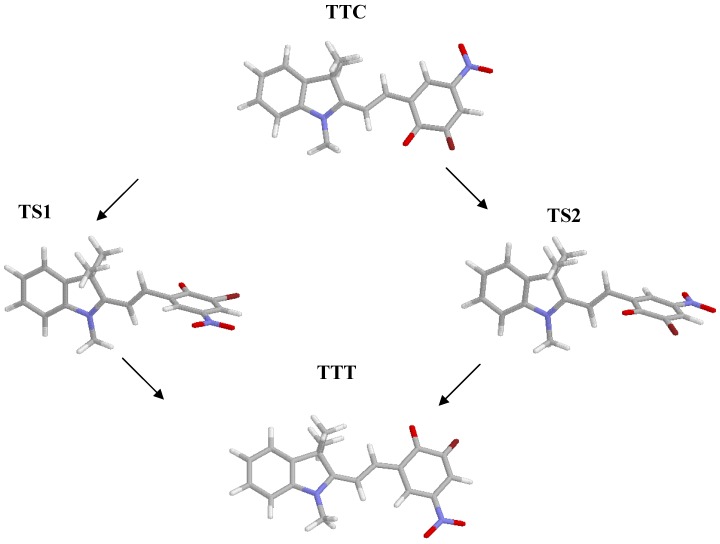
Optimized structures of TTC, TTT and of the transition states.

Given the absence of symmetry of the isomers of the system considered, because of the presence of the methyl groups, two transitions states of the TTC➔TTT isomerization reaction have to be found, depending on the rotation, clockwise or counter-clockwise, of the benzene moiety around the C10-C11 bond. The energy values obtained in gas phase and in dichloromethane, along with the most significant dihedral angles and bond lengths of the two transition states, are shown in Table 2; the optimized structures are depicted in Figure 2. 

**Table 2:** Relative energies (kJ·mol^-1^) and relevant torsional angles (degrees) and bond lengths (Å) of the transition states in the isomerization reaction TTC→TTT of 8-bromo- 6-nitro-BIPS in *vacuo* and in methylene chloride solution at different temperatures. Zero energy reference values are those of the TTC isomer.

	6-NO_2_-8-Br-BIPS *in vacuo*		6-NO_2_-8-Br-BIPS in CH_2_Cl_2_ solution	
	TS1	TS2	TS1	TS2
**E**	125.0	126.1	70.9	70.9
**H_190_**	120.3	119.5	66.2	65.9
**G_190_**	120.6	122.9	72.5	72.8
**H_298_**	119.8	118.5	64.6	64.6
**G_298_**	120.8	125.3	76.4	76.7
**α**	-174.5	178.6	-178.1	-178.0
**β**	-170.1	173.5	-179.5	179.5
**γ**	86.3	-87.1	85.9	-85.4
**C8-C10**	1.420	1.417	1.436	1.436
**C10-C11**	1.363	1.364	1.354	1.354
**C11-C12**	1.473	1.476	1.482	1.482

An increase of the C8-C10 and C11-C12 bond lengths is observed when going from the ground states to the transition states; this effect is more pronounced in the presence of the solvent, in agreement with the increased role of the resonance structure involving charge separation (N^+^, O^-^) in a polar solvent.

With regards to the dihedral angles, the value of β is lower in the transition state in *vacuo* whereas it is essentially the same as in the ground states when the solvent is considered. The theoretical activation enthalpy, in CH_2_Cl_2_ solution, averaged over the temperature, is about 66 kJ∙mol^-1^. Further single point calculations performed by adding diffuse functions (6-31++G(d,p) basis set) give an activation energy value of about 57 kJ·mol^-1^. This value should be strictly compared with the experimental value obtained by the Arrhenius plot, 43.6±3 kJ∙mol^-1^ [12]. 

To the best of our knowledge, this is the best theoretical evaluation of the activation energy of the TTC→TTT isomerization in solution. In fact, a calculation of the activation energy for the same reaction, by considering the same isomers *in vacuo* without methyl groups and bromine, provided a value of 139 kJ∙mol^-1^ [16]. This clearly indicates the importance of considering the substituent groups (methyls and bromine), whose presence decreases the activation energy value *in vacuo* by about 13 kJ∙mol^-1^ (see Table 2), but mainly points out the large solvent effect, which almost halves the activation energy value calculated *in vacuo*.

By examining the geometrical parameters of the transition states when going from gas phase to the solution, it is possible to correlate the solvent effects on the activation energy with the transition state geometries. In our case, there is a significant increasing of the value of the β dihedral angle when going from gas phase to CH_2_Cl_2_ solution, the value in solution being very similar to that observed in the ground states of the merocyanines. This effect seems responsible for the large decreasing of the activation energy in solution. The influence of the solvent on the geometry of the transition state was already observed in some other reactions in organic chemistry, as in the case of the decarboxylation reaction [17].
